# Atypical Presentation of Rapidly Progressive Guillain–Barré Syndrome With Dysautonomia in an Adolescent With Persistent *Mycoplasma pneumoniae* Infection: A Case Report

**DOI:** 10.1155/crpe/5766838

**Published:** 2026-06-30

**Authors:** Madhu Parna Chakrabarti, Richard Sultan, Daniel Ruderfer, Katherine Hickey

**Affiliations:** ^1^ St. George’s University School of Medicine, Great River, New York, USA, sgu.edu; ^2^ Department of Pediatric Neurology, HMH Jersey Shore University Medical Center, Neptune City, New Jersey, USA; ^3^ Department of Pediatric Infectious Diseases, HMH Jersey Shore University Medical Center, Neptune City, New Jersey, USA; ^4^ Department of Pediatric Critical Care Medicine, HMH Jersey Shore University Medical Center, Neptune City, New Jersey, USA

**Keywords:** AIDP, dysautonomia, Guillain–Barré syndrome, miller fisher syndrome, *Mycoplasma pneumoniae*, pharyngeal–cervical–brachial variant

## Abstract

**Background:**

Guillain–Barré syndrome (GBS) is an acute immune‐mediated polyradiculoneuropathy and the leading cause of acute flaccid paralysis in children. *Mycoplasma pneumoniae* is a frequent antecedent infection in pediatric GBS and has been associated with a severe, rapidly progressive course with heterogeneous neurologic manifestations, creating diagnostic and therapeutic challenges. We report here an atypical case of rapidly progressive GBS in an adolescent with persistent *M. pneumoniae* infection.

**Case Report:**

A previously healthy 17‐year‐old female presented with rapidly progressive neurological deficits, including asymmetric proximal flaccid quadriparesis, hypophonia, absent gag reflex, and hyporeflexia, following *M. pneumoniae* infection treated with azithromycin. MRI and electrodiagnostic studies supported acute inflammatory demyelinating polyradiculoneuropathy. Despite intravenous immunoglobulin (IVIG), she deteriorated with bilateral facial palsy, ophthalmoplegia, dysautonomia, and respiratory failure due to persistent polymicrobial pneumonia requiring mechanical ventilation. The fulminant course showed overlapping features of multiple GBS variants. She received additional IVIG and subsequent plasmapheresis, resulting in gradual neurological improvement. She was discharged to inpatient rehabilitation following ventilator weaning with residual limb weakness and neurogenic bladder.

**Conclusion:**

This case underscores the diagnostic and therapeutic challenges of atypical, rapidly progressive GBS in pediatric patients with persistent *M. pneumoniae* infection and overlapping clinical features of multiple GBS variants. Concomitant dysautonomia, acute respiratory failure, and lack of response to IVIG further emphasize the importance of early recognition and meticulous supportive care. Given that *M. pneumoniae* is a leading trigger of severe pediatric GBS, clinicians should maintain a high index of suspicion and promptly initiate and adapt established therapies to optimize outcomes.

## 1. Introduction

Guillain–Barré syndrome (GBS) is an acute, immune‐mediated polyradiculoneuropathy that classically presents with progressive, symmetrical weakness and areflexia. Although the incidence of GBS increases with advancing age, it remains the most common cause of acute flaccid paralysis in the pediatric population [1].

GBS is typically preceded by an infectious illness or other immune stimulus that triggers an aberrant autoimmune response directed against peripheral nerves and spinal roots [2, 3]; however, its precise pathogenesis has yet to be fully elucidated [3, 4]. Both cellular and humoral immune mechanisms have been implicated, with evidence suggesting the involvement of nearly all components of the immune system [5]. In a subset of patients, circulating serum antibodies directed against gangliosides are detectable. Pathological features of affected peripheral nerves and nerve roots commonly include complement activation, macrophage infiltration, and endoneurial edema [3]. Molecular mimicry between microbial antigens and neural components is recognized as a central mechanism in the pathogenesis of the disorder [4, 6]. A wide range of infectious agents have been implicated as triggers of GBS, including Epstein–Barr virus, *cytomegalovirus*, *varicella*, *Campylobacter jejuni*, and *Mycoplasma pneumoniae* [7].

*M. pneumoniae*–associated GBS occurs more frequently in children than in adults and is characterized by the generation of antibodies against bacterial surface glycolipids, particularly galactocerebroside (GalC); anti‐GalC IgG antibodies have been proposed to contribute to disease pathogenesis [8]. Epidemiologically, *M. pneumoniae* accounts for approximately 21% of the pediatric GBS cases compared with only 3% in adults [8]. Although most cases of *M. pneumoniae*–related GBS follow a monophasic, postinfectious course, atypical presentations, especially in pediatric patients, may be rapid in onset and severe in progression. Consistent with this observation, accumulating evidence supports an association between *M. pneumoniae* infection and severe, rapidly progressive forms of GBS [9].

Patients with GBS typically present with ascending weakness accompanied by sensory disturbances that begin in the lower extremities and progress to involve the upper limbs and cranial nerves. This pattern is characteristic of acute inflammatory demyelinating polyradiculoneuropathy (AIDP), the most prevalent GBS subtype [5]. Nonetheless, GBS encompasses several well‐recognized variants with distinct clinical and pathological features [5]. Particularly in pediatric and adolescent populations, neurologic presentations are frequently heterogeneous and variable [3, 5]. In this report, we describe an atypical, rapidly progressive presentation of GBS in an adolescent female with persistent *M. pneumoniae* infection, underscoring the associated diagnostic and therapeutic challenges.

## 2. Case Report

A 17‐year‐old previously healthy adolescent female presented to the pediatric emergency department with a two‐week history of cough, nasal congestion, and sore throat, accompanied by 10 days of progressive weakness that initially involved the lower extremities and subsequently ascended to involve the upper extremities. Prior to presentation, she was evaluated at an urgent care facility, where testing for Group A streptococcus, SARS‐CoV‐2, and influenza was negative; she was empirically treated with amoxicillin without clinical improvement. At presentation, she denied fever, chills, neck pain, photophobia, bulbar symptoms, or bowel or bladder incontinence. Additionally, the patient reported no tobacco, alcohol, or illicit substance use. She was admitted to the general pediatric service for further neurologic evaluation.

**FIGURE 1:**
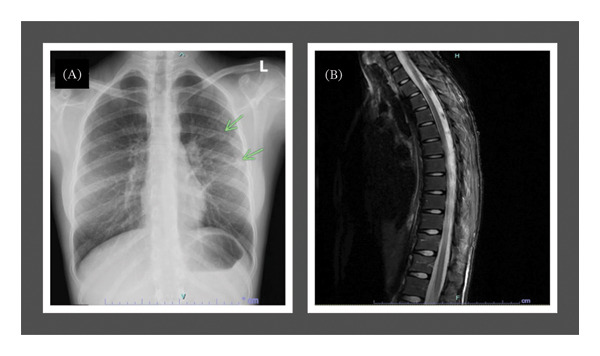
(A) X‐ray chest PA and lateral done on the day of admission showing patchy left upper lobe infiltrate; no pleural effusion or pneumothorax noted. (B) MRI thoracic spine with or without contrast performed on HD 8 demonstrating the enhancement of both the ventral and caudal nerve roots of the mid to lower thoracic spine. There also appears to be enhancement of the cauda equina nerve roots. No lumbar spine MRI was performed.

On admission, physical examination revealed maxillary sinus tenderness and decreased breath sounds over the left lung fields. Laboratory studies were largely unremarkable, aside from mild leukocytosis with neutrophil predominance, thrombocytosis, and mildly elevated C‐reactive protein (Table 1A). Chest radiography demonstrated patchy infiltrates in the left upper lobe (Figure 1A), and sputum polymerase chain reaction (PCR) was positive for *M. pneumoniae*. She was diagnosed with atypical pneumonia and dehydration and treated with intravenous fluids and azithromycin.

**TABLE 1:** Clinical laboratory values of blood and cerebrospinal fluid.

A: Blood^1^			
Tests	Result	Reference ranges	—
Hgb	13.3	12.0–16.0 g/dL	
RBC	4.69	4.10–5.10 10^6^/μL	
WBC	12.5	4.5–11.0 10^3^/μL	
ANC	10.6	1.8–8.0 10^3^/μL	
ALC	1.4	1.5–3.5 10^3^/μL	
Platelet count	461	140–450 10^3^/μL	
Sodium	138	136–145 mmol/L	
Potassium	3.6	3.5–5.1 mmol/L	
Chloride	100	98–107 mmol/L	
Glucose	119	70–99 mg/dL	
AST	21	0–34 U/L	
ALT	18	10–49 U/L	
Total bilirubin	0.9	0.2–1.3 mg/dL	
BUN	9	9–23 mg/dL	
Cr	0.59	0.55–1.02 mg/dL	
CRP	1.2	< 0.5 mg/dL	
Procalcitonin	0.05	< 0.50 ng/mL	
MOG Ab Screen	Positive^∗^	Negative	

B: Cerebrospinal fluid**^2^**			

**Tests**	**HD 1**	**HD 8**	**Reference ranges**

Appearance	Clear	Clear	Clear
Color	Colorless	Straw	Colorless
Glucose	64.3	85.6	60.0–80.0 mg/dL
Protein	80.6	394.0	15.0–45.0 mg/dL
WBC	1	4	0–5/μL
Xanthochromia	No	Yes	No
MOG Ab	N/A	Negative^∗∗^	Negative
CSF culture and Gram stain	No growth	No growth	No growth

^1^Diagnostic workups were performed on the day of admission.

^2^Lumbar puncture and CSF analysis were performed on HD 1 and HD 8.

^∗^MOG Ab Screen was done on HD 2.

^∗∗^MOG Ab was done only on HD 8.

Over the subsequent 24 hours, the patient experienced rapid neurologic deterioration and was transferred to the pediatric intensive care unit (PICU). On arrival at the PICU, examination revealed hypophonia, absence of the gag reflex, and asymmetric, predominantly proximal weakness. Motor strength was graded as 1–2/5 in the proximal lower extremities and 4/5 distally, proximal upper extremity as 1/5 on the left and 3/5 on the right. Deep tendon reflexes were absent at the knees, diminished (1+) at the ankles, biceps, and brachioradialis, with preserved plantar reflexes. Given concern for imminent respiratory failure, the patient was intubated for airway protection. Since her weakness was asymmetric, a magnetic resonance imaging (MRI) was performed to rule out any intracranial pathology. MRI brain and spine were initially normal. A lumbar puncture performed on hospital day (HD) 1 demonstrated albuminocytologic dissociation, with an elevated cerebrospinal fluid (CSF) protein (80.6 mg/dL) and normal CSF white blood cell count (Table 1B). Serum screening was initially positive for myelin oligodendrocyte glycoprotein (MOG) IgG, though CSF was negative for MOG antibodies (Table 1A and Table 1B). Serum antiganglioside antibody titer was not performed. Given the concern for GBS, the patient was initiated on intravenous immunoglobulin (IVIG) at 400 mg/kg/day starting HD 2 for a planned 5‐day course.

Despite treatment, the patient experienced progressive neurological deterioration. By HD 3, she developed worsening weakness with flaccid paralysis of the lower extremities, new bilateral facial palsies with full abduction of the right eye. On HD 5, severe dysautonomia emerged with profound swings in blood pressure and heart rate including persistent systolic hypertension in the 180 s mmHg and tachycardia with heart rates in the 130s, necessitating the initiation of a continuous nicardipine infusion. Renal and bladder ultrasonography with Doppler revealed normal morphology and no evidence of renal artery stenosis. By HD 6, the patient had progressed to quadriplegia.

Following completion of the initial 5‐day IVIG course, there was no appreciable neurological improvement. MRI of the spine with contrast, performed on HD 8, demonstrated enhancement of the ventral and caudal nerve roots involving the mid‐to‐lower thoracic spine and cauda equina (Figure 1B). A repeat lumbar puncture on HD 8 revealed a marked increase in CSF protein to 394 mg/dL (Table 1B). Although plasmapheresis was initially planned, refractory hypertension precluded immediate initiation, and the patient instead received an additional 2 days of IVIG.

With subsequent blood pressure stabilization, plasmapheresis commenced on HD 10, with completion of five sessions over 10 days. By HD 12, she began to exhibit some neurological improvement, including return of the gag reflex, mild improvement in facial strength, and a stable isolated left cranial nerve VI palsy. However, she continued to have global areflexia and flaccid paralysis of all four extremities. By HD 24, the patient demonstrated substantial neurological recovery, with full extraocular movements, strong cough and gag reflexes, near‐complete resolution of facial palsies, and return of muscle strength in the proximal lower extremities as well as distal upper extremities and digits.

**FIGURE 2:**
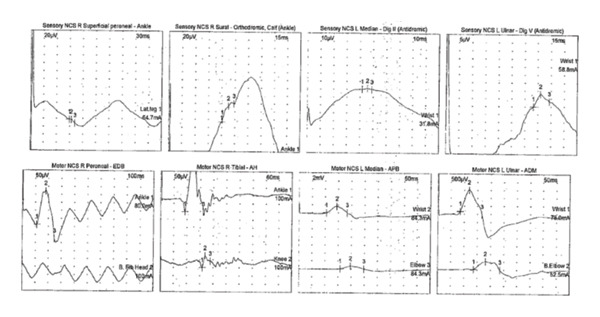
Electrodiagnostic studies performed on HD 26 are consistent with diffuse demyelinating neuropathy with conduction block and relative sparing of the right sural nerve. Needle electromyography showed no abnormal insertional activity and no evidence of significant axonal involvement.

Electrodiagnostic studies performed on HD 26 were consistent with a demyelinating sensorimotor neuropathy with conduction block and relative sparing of the right sural nerve. Needle electromyography showed no abnormal insertional activity and no evidence of significant axonal involvement (Table 2 and Figure 2).

**TABLE 2:** Nerve conduction studies performed on HD 26 demonstrates data consistent with diffuse demyelinating neuropathy.

A: Sensory nerve conduction study									
Nerve/Sites	Rec. Site	Onset lat (ms)	Peak lat (ms)	NP amp (μV)	PP amp (μV)	Segments	Distance (cm)	Velocity (m/s)	—
*L Median–Dig II (Antidromic)*									
Wrist	Index	4.17	4.56	1.1	1.6	Wrist–Index	14	34	

*L Ulnar–Dig V (Antidromic)*									
Wrist	Dig V	9.96	10.79	8.0	4.7	Wrist–Dig V	14	14	

*R Superficial Peroneal–Ankle*									
Lat leg	Ankle	8.94	9.38	0.23	8.9	Lat leg–Ankle	14	16	

*R Sural–Orthodromic, Calf (Ankle)*									
Ankle	Calf	6.08	6.77	40.2	5.7	Ankle–Calf	14	23	

**B: Motor nerve conduction study**									

**Nerve/sites**	**Muscle**	**Latency (ms)**	**Amplitude (mV)**	**Rel Amp (%)**	**Segments**	**Distance (cm)**	**Lat Diff (ms)**	**Velocity (m/s)**	**Rel Vel (%)**

*L Median–APB*									
Wrist	APB	10.42	1.6	—	Wrist–APB	8	—	—	—
Elbow	APB	15.81	0.7	45.1	Elbow–Wrist	18.7	5.40	35	100

*L Ulnar–ADM*									
Wrist	ADM	8.46	1.4	100	Wrist–ADM	8	—	—	—
Below elbow	ADM	13.65	0.5	37.1	B. Elbow–Wrist	24.8	5.19	48	100

*R Peroneal–EDB*									
Ankle	EDB	11.17	0.2	100	Ankle–EDB	8	—	—	—
B. Fib Head	EDB	—	—	—	B. Fib Head–Ankle	—	—	—	—

*R Tibial–AH*									
Ankle	AH	11.04	0.4	100	Ankle–AH	8	—	—	—
Knee	AH	19.13	0.1	21.6	Knee–Ankle	34.7	8.08	43	100

*Note:* A. The sensory nerve conduction studies demonstrate prolonged sensory onset latencies and reduced SNAP amplitudes and slowed conduction velocities with relative sparing in the sural nerve. B. The motor nerve conduction studies demonstrate prolonged latencies, significantly reduced CMAP amplitudes and near normal conduction velocities in the lower limbs and normal conduction velocities in the upper limbs.

The patient’s hospital course was complicated by ventilator‐associated pneumonia. Bronchoalveolar lavage, performed for the clearance of deep secretions, resulted in cultures positive for *Pseudomonas aeruginosa* and methicillin‐sensitive *Staphylococcus aureus* (MSSA). Persistent infection with *M. pneumoniae* was also detected by PCR despite prior macrolide therapy. Antimicrobial therapy was reinitiated and included doxycycline to cover persistent *M. pneumoniae*, ciprofloxacin for *P. aeruginosa*, and cefazolin for MSSA. The patient responded positively to antibiotic therapy with improvement of oxygen requirement and ventilator pressures back to baseline. At this time, extubation was attempted; however, she quickly required reintubation for hypercarbic respiratory failure in the setting of persistent neuromuscular weakness.

At the time of discharge on HD 33, the patient was able to maintain upright head and neck posture, demonstrated full strength of the facial, head, and neck musculature, and was able to shrug her shoulders. She exhibited partial recovery of motor function with limited pronation and supination of the bilateral upper and lower extremities with emerging grip strength. Ventilator support was progressively weaned, and she tolerated prolonged spontaneous breathing trials. She continued to require intermittent bladder catheterization for neurogenic bladder secondary to dysautonomia. She was discharged to an acute inpatient rehabilitation facility with a tracheostomy in place.

## 3. Discussion

GBS is an immune‐mediated inflammatory disorder of the peripheral nervous system and the leading cause of acute flaccid paralytic neuropathy. It comprises a heterogeneous group of clinically and pathologically distinct variants. AIDP, the most common subtype, typically presents with rapidly progressive, ascending, symmetric limb weakness, areflexia, and sensory disturbances, reaching maximal severity within 2‐3 weeks [5]; acroparesthesia with minimal objective sensory loss is often the earliest symptom [10]. Acute motor axonal neuropathy (AMAN) is an axonal variant characterized by rapidly progressive, severe pure motor paralysis with minimal or absent sensory involvement [5, 11], whereas acute motor and sensory axonal neuropathy (AMSAN) features profound weakness, sensory loss, and areflexia due to axonal degeneration rather than demyelination [5, 12]. Compared with AIDP, AMAN typically reaches nadir more rapidly, often within days, and is associated with prolonged paralysis and early respiratory failure [13]. AMAN is strongly associated with serum IgG antibodies against gangliosides GD1a and GM1 [14], with anti‐GD1a IgG detected in approximately 60% of the cases compared with 4% in AIDP [15].

Miller Fisher syndrome is an uncommon variant of GBS, defined by the acute onset of the characteristic triad of ophthalmoplegia, ataxia, and areflexia, typically without accompanying limb weakness [5, 16]. In addition to this classic presentation, several less frequent phenotypic variants have been described, including forms with predominant autonomic or regionally restricted involvement. One such variant, the pharyngeal–cervical–brachial subtype, accounts for up to 3% of the cases and is characterized by ptosis and weakness involving the facial, pharyngeal, and neck flexor muscles, with relative preservation of lower limb strength, sensation, and reflexes [5].

In this case report, we describe a 17‐year‐old adolescent female who presented with a rapidly progressive GBS in the setting of persistent *M. pneumoniae* infection, manifesting an atypical clinical phenotype that does not conform to the classic features of previously described GBS variants. Electrodiagnostic studies demonstrated a demyelinating sensorimotor neuropathy without evidence of significant axonal involvement, findings consistent with AIDP (Table 2 and Figure 2). In addition, contrast‐enhanced MRI demonstrated enhancement of both the ventral and caudal nerve roots of the mid‐to‐lower thoracic spine and the signal of enhancement of the cauda equina, radiographic features commonly observed in AIDP (Figure 1B) [17].

Acute flaccid myelitis (AFM) is a rare but serious neurological condition characterized by sudden flaccid muscle weakness and loss of reflexes, predominantly in pediatric patients. AFM is typically triggered by viral infections, including Enteroviruses, West Nile Virus, and Adenoviruses, and is commonly associated with lymphocytic predominance in the CSF [18]. Additionally, electromyography and nerve conduction studies in AFM typically demonstrate preserved sensory nerve action potentials [19], while MRI characteristically reveals nonenhancing, multilevel spinal cord lesions [18]. These findings were not present in this patient, which ruled out the differential diagnosis of AFM.

The patient’s neurological status deteriorated rapidly within 24 hours, with an asymmetric pattern of weakness that predominantly involved proximal muscle groups of both the upper and lower extremities and progressed to quadriplegia by HD 6. The clinical course was further complicated by early respiratory failure resulting in a prolonged and severe disease course. This fulminant progression is more typically associated with AMAN rather than AIDP. The patient also developed bilateral facial palsies accompanied by ophthalmoplegia, including impaired abduction of the right eye on HD 3. These clinical features are not characteristic of classic AIDP but are more frequently reported in less common GBS variants, such as Miller Fisher syndrome, characterized by ophthalmoplegia and areflexia, and the pharyngeal–cervical–brachial variant, in which facial weakness is prominent. However, both Miller Fisher syndrome and the pharyngeal–cervical–brachial variant typically spare significant limb weakness, which was the dominant presenting and progressive feature in this patient.

The patient’s neurological course was further complicated by dysautonomia causing cardiac dysfunction; she also developed neurogenic bladder secondary to dysautonomia, possibly affecting both peripheral parasympathetic and somatic nerves [20]. Dysautonomia is reported in approximately two‐thirds of the patients with GBS and occurs with increased frequency in severe pediatric cases, including those associated with *M. pneumoniae* infection [17, 21]. Although autonomic dysfunction does not directly extend the duration of *M. pneumoniae* infection, severe dysautonomia in GBS may hinder airway clearance and predispose patients to secondary complications, thereby contributing to persistent respiratory infection in intubated individuals. In the present case, the patient experienced an acute deterioration marked by pneumonia with confirmed polymicrobial infection, including *P. aeruginosa* and MSSA. These findings underscore the multifactorial interaction between impaired host defenses, the need for mechanical ventilation, secondary bacterial colonization, and dysautonomia [21, 22]. Additionally, persistent *M. pneumoniae* was detected by PCR despite prior macrolide therapy and may have further exacerbated respiratory compromise, ultimately resulting in acute hypoxic respiratory failure necessitating prolonged mechanical ventilation.

The standard treatment for GBS consists of IVIG administered at a cumulative dose of 2 g/kg over a 5‐day period [5]. In neuromuscular disorders, IVIG is thought to exert its therapeutic effects through multiple mechanisms, including disruption of complement activation and membrane attack complex formation, interference with costimulatory pathways involved in antigen presentation, modulation of autoantibody and cytokine activity, and regulation of macrophage Fc receptor function [23]. In the present case, the patient did not demonstrate meaningful neurological improvement following the initial 5‐day course of IVIG. Plasmapheresis was initially planned; however, refractory hypertension precluded its initiation, prompting administration of an additional 2 days of IVIG. Early neurological improvement was subsequently observed after completion of this second course. Notably, a study from the Netherlands reported that patients with GBS at high risk for poor outcomes may derive benefit from a second IVIG course administered shortly after the first [24, 25]. Although five sessions of plasmapheresis were later performed following stabilization of blood pressure, it remains unclear whether this intervention conferred additional benefit to the patient’s recovery. Consistent with prior evidence, combination therapy with IVIG and plasmapheresis has not been shown to provide a significant advantage over either treatment alone [26].

## 4. Conclusion

This case highlights the diagnostic and therapeutic complexities of an atypical, rapidly progressive GBS in an adolescent with persistent *M. pneumoniae* infection. The patient’s extensive and heterogeneous neurologic manifestations created substantial challenges in delineating a specific GBS variant, emphasizing the difficulty of diagnosing and managing pediatric GBS when the clinical course is variable. Concomitant involvement of the autonomic nervous system further complicated management of the fulminant presentation, as cardiac dysfunction and blood pressure instability are recognized contributors to mortality, which remains estimated at 3%–10% in pediatric GBS despite optimal medical therapy [27–29]. In addition, the persistent *M. pneumoniae* infection contributed to a complicated hospital course, further underscoring the complexity of care in such cases. Given the broad spectrum of GBS presentations, clinicians should maintain a high index of suspicion and be prepared to adjust management strategies in response to evolving clinical and diagnostic findings. Early recognition and timely initiation of established therapies remain critical to optimizing patient outcomes.

## Funding

The authors have nothing to report.

## Consent

Informed consent was received from all patients.

## Conflicts of Interest

The authors declare no conflicts of interest.

## Data Availability

The data that support the findings of this study are available on request from the corresponding author. The data are not publicly available due to privacy or ethical restrictions.
